# An integrated transcriptomics and proteomics analysis reveals functional endocytic dysregulation caused by mutations in *LRRK2*

**DOI:** 10.1016/j.nbd.2019.04.005

**Published:** 2019-07

**Authors:** Natalie Connor-Robson, Heather Booth, Jeffrey G. Martin, Benbo Gao, Kejie Li, Natalie Doig, Jane Vowles, Cathy Browne, Laura Klinger, Peter Juhasz, Christine Klein, Sally A. Cowley, Paul Bolam, Warren Hirst, Richard Wade-Martins

**Affiliations:** a Oxford Parkinson's Disease Centre, Department of Physiology, Anatomy and Genetics, University of Oxford, Oxford, UK; b Biogen, Cambridge, MA, USA; c Medical Research Council Brain Network Dynamics Unit, University of Oxford, Oxford, UK; d James Martin Stem Cell Facility, Sir William Dunn School of Pathology, University of Oxford, South Parks Road, Oxford OX1 3RE, UK.; e Institute of Neurogenetics, University of Leubeck, Maria-Goeppert-Str. 1, 23562 Luebeck, Germany.; f Oxford Parkinson's Disease Centre (OPDC), Oxford, UK

**Keywords:** LRRK2, Parkinson's disease, Endocytosis, Clathrin, Endophilin, Rabs, PD, Parkinson's Disease, SNpc, Substantia Nigra pars compacta, CME, Clathrin-meadiated endocytosis, iPSC, induced pluripotent stem cell, EM, Electron microscopy, PCA, Principal component analysis, FDR, False-discovery rate, EGSEA, Ensemble of Gene set enrichment analyses, hWT, LRRK2 human wild-type, nTG, non-transgenic, SVs, synaptic vesicles

## Abstract

**Background:**

Mutations in *LRRK2* are the most common cause of autosomal dominant Parkinson's disease, and the relevance of *LRRK2* to the sporadic form of the disease is becoming ever more apparent. It is therefore essential that studies are conducted to improve our understanding of the cellular role of this protein. Here we use multiple models and techniques to identify the pathways through which *LRRK2* mutations may lead to the development of Parkinson's disease.

**Methods:**

A novel integrated transcriptomics and proteomics approach was used to identify pathways that were significantly altered in iPSC-derived dopaminergic neurons carrying the *LRRK2-G2019S* mutation. Western blotting, immunostaining and functional assays including FM1-43 analysis of synaptic vesicle endocytosis were performed to confirm these findings in iPSC-derived dopaminergic neuronal cultures carrying either the *LRRK2-G2019S* or the *LRRK2-R1441C* mutation, and *LRRK2* BAC transgenic rats, and post-mortem human brain tissue from *LRRK2-G2019S* patients.

**Results:**

Our integrated -omics analysis revealed highly significant dysregulation of the endocytic pathway in iPSC-derived dopaminergic neurons carrying the *LRRK2-G2019S* mutation. Western blot analysis confirmed that key endocytic proteins including endophilin I-III, dynamin-1, and various RAB proteins were downregulated in these cultures and in cultures carrying the *LRRK2-R1441C* mutation, compared with controls. We also found changes in expression of 25 RAB proteins. Changes in endocytic protein expression led to a functional impairment in clathrin-mediated synaptic vesicle endocytosis. Further to this, we found that the endocytic pathway was also perturbed in striatal tissue of aged *LRRK2* BAC transgenic rats overexpressing either the *LRRK2* wildtype, *LRRK2-R1441C* or *LRRK2-G2019S* transgenes. Finally, we found that clathrin heavy chain and endophilin I-III levels are increased in human post-mortem tissue from *LRRK2-G2019S* patients compared with controls.

**Conclusions:**

Our study demonstrates extensive alterations across the endocytic pathway associated with *LRRK2* mutations in iPSC-derived dopaminergic neurons and BAC transgenic rats, as well as in post-mortem brain tissue from PD patients carrying a LRRK2 mutation. In particular, we find evidence of disrupted clathrin-mediated endocytosis and suggest that LRRK2-mediated PD pathogenesis may arise through dysregulation of this process.

## Highlights

• iPSC-derived dopaminergic neurons from *LRRK2* patients show extensive endocytic changes.
• Integrated proteomic and transcriptomic approach reveals dysregulation of 25 RABs.
• Functional impairment of clathrin mediated endocytosis in *LRRK2* iPSC-dopaminergic neurons.
• Aged *LRRK2* rats also show similar perturbations of key endocytic proteins.
• *LRRK2* human post-mortem tissue shows upregulation of clathrin and endophilin.

## 1 Introduction

Parkinson's disease (PD) is a common neurodegenerative disorder in which dopaminergic neurons of the *substantia nigra pars compacta* (SNpc) are lost, leading to the classic motor symptoms of the disease. Mutations in *LRRK2* are the most common cause of late-onset autosomal dominant forms of PD and are clinically indistinguishable from sporadic cases (Funayama et al., 2005; Zimprich et al., 2004). Single nucleotide polymorphisms at the *LRRK2* locus have also been identified as PD risk factors through genome wide association studies and have recently been shown to lead to increased kinase activity in the sporadic disease (Chang et al., 2017; Di Maio et al., 2018; Nalls et al., 2014). The LRRK2 protein contains both kinase and GTPase domains as well as regions involved in protein-protein interactions. Multiple *LRRK2* mutations have been associated with PD; these include the G2019S mutation in the kinase domain of the protein, and the R1441C mutation in the GTPase domain.

Clathrin-mediated endocytosis (CME) is an important cellular function required for the recycling of synaptic vesicles and certain plasma membrane components (Saheki and De Camilli, 2012). Neurons can undergo periods of extensive synaptic transmission which is highly dependent on the fast and efficient recycling of synaptic vesicles. Previous work has shown that LRRK2 fractionates with important synaptic proteins such as synapsin, synaptophysin, NSF, dynamin-1, and VAMP2 (Arranz et al., 2014; Belluzzi et al., 2016; Carrion et al., 2017; Piccoli et al., 2014). Further to this LRRK2 has been shown to directly interact with some key endocytic components including endophilin, dynamin and auxilin (Matta et al., 2012; Nguyen and Krainc, 2018; Piccoli et al., 2014). However, the extent to which mutations in *LRRK2* alter the process of CME in dopaminergic neurons remains poorly described.

Here we present data from an integrated proteomic and transcriptomic analysis of induced-pluripotent stem cell (iPSC)-derived dopaminergic cultures from PD patients carrying the *LRRK2-G2019S* mutation, which reveals dysregulation of endocytosis in these cells. We then demonstrate that levels of the endocytic proteins clathrin and endophilin are reduced in iPSC-derived dopaminergic cultures from *LRRK2-G2019S* and *LRRK2-R1441C* mutation carriers, as well as in 22-month-old *LRRK2* BAC transgenic rats carrying these same mutations. Furthermore, we identify clear changes in the levels of key RAB proteins in both models and demonstrate the detrimental functional impact of *LRRK2* mutations on CME in iPSC-derived dopaminergic neurons. Finally, we demonstrate that clathrin and endophilin are both dysregulated in post mortem striatal brain tissue from PD patients carrying the *LRRK2-G2019S* mutation. Together, these findings demonstrate that *LRRK2* mutations lead to perturbations in CME, and present a plausible mechanism for the development of PD pathogenesis.

## 2 Materials and methods

### 2.1 Participant recruitment and *LRRK2* mutation screening

Participants were recruited through the Oxford Parkinson's Disease Centre Discovery clinical cohort and gave signed informed consent to mutation screening and derivation of iPSC lines from skin biopsies (Ethics committee: National Health Service, Health Research Authority, NRES Committee South Central, Berkshire, UK, REC 10/H0505/71). All UK patients fulfilled the UK Brain Bank diagnostic criteria for clinically probable PD at presentation. Healthy controls were age-matched to within a decade where possible.

### 2.2 Culture and reprogramming of primary fibroblasts

Low passage fibroblast cultures were established from participant skin punch biopsies and reprogrammed using the CytoTune-iPSC Sendai Reprogramming kit (Invitrogen). JR207-3 was reprogrammed using an alternative Sendai system SeVdp(KOSM)mir301L, which contains a target for mir302, which is expressed in pluripotent cells, but not in the originating fibroblasts, ensuring effective removal of exogenous genetic material within a few passages. Retrovirally reprogrammed lines used in this study had been reprogrammed using Yamanaka reprogramming vectors, as described previously (Hartfield et al., 2012). Clones were transitioned from initial culture on mitotically inactivated CF1 Mouse Embryonic Feeders (Millipore) to feeder-free culture in mTeSR™ medium (StemCell Technologies), on hESC-qualified Matrigel-coated plates (BD).

Characterization of previously unpublished iPSC lines was performed on bulk frozen stocks (Fig. S2). Briefly, flow cytometry for pluripotency markers TRA-1-60 (Biolegend #33061, Alexa Fluor 488), and Nanog (Cell Signalling #5448, Alexa Fluor 647) was performed on a FACSCalibur (BD Biosciences).

For Cytotune 1 Sendai virus clearance, the following primers were used for standard PCR:

Sendai virus forward: GGATCACTAGGTGATATCGAGC

Sendai virus reverse: ACCAGACAAGAGTTTAAGAGATATGTATC

SOX2 forward: ATGCACCGCTACGACGTGAGCGC

SOX2 reverse: AATGTATCGAAGGTGCTCAA

KLF4 forward: TTCCTGCATGCCAGAGGAGCCC

KLF4 reverse: AATGTATCGAAGGTGCTCAA

c-MYC forward: TAACTGACTAGCAGGCTTGTCG

c-MYC reverse: TCCACATACAGTCCTGGATGATGATG

OCT4 forward: CCCGAAAGAGAAAGCGAACCAG

OCT4 reverse: AATGTATCGAAGGTGCTCAA

For Cytotune 2, Sendai, KLF and MYC were as for Cytotune 1, and the KLF4/OCT4/SOX2 construct was detected using primers:

KOS (KLF/OCT4/SOX2) forward: ATG CAC CGC TAC GAC GTG AGC GC

KOS (KLF/OCT4/SOX2) reverse: ACC TTG ACA ATC CTG ATG TGG.

The β-Actin qPCR Control Kit (Eurogentec) was used as control for normalization.

Genome integrity and cell identity tracking used the Human CytoSNP-12v2.1 beadchip array or OmniExpress24 array (Illumina) on genomic DNA (All-Prep kit, Qiagen) analysed with GenomeStudio and Karyostudio software (Illumina).

### 2.3 Maintenance of iPSC lines

iPSCs were maintained as colonies in mTesR1 medium (Stem Cell Technologies) supplemented with Penicillin (100 U/mL)-Streptomycin (100 μg/mL) (1× PenStrep) (ThermoFisher Scientific) and passaged once confluent using 500 μM EDTA.

### 2.4 Differentiation of iPSCs to dopaminergic neurons

Differentiation of iPSCs was carried out as previously described (Beevers et al., 2017), with the minor modification that, at day 20 of the protocol, cells were plated at 5 × 10^5^/cm^2^. Cells were maintained in final medium as previously described until 35 or 56 DIV.

### 2.5 Genotyping of iPSC lines

Genomic DNA was extracted from iPSCs using Illustra Tissue and Cells Genomic Miniprep Kit (GE Healthcare. PCR was carried out using AmpliTaq Gold DNA polymerase with primers specific for regions of the LRRK2 gene that contain the G2019S (5’-TTTAAGGGACAAAGTGAGCAC-3′ and 5′-ACTCTGTTTTCCTTTTGACTC-3′) and R1441C (5’-GGAGGACCCAATTGGGTGCGT-3′ and 5′-ACGCTGTCTTCAGCCCACTTC-3′) mutations. PCR products were digested using *Sfc*I (G2019S) or *Bst*UI (R1441C) restriction enzymes and products analysed for the presence of mutations by agarose gel electrophoresis.

### 2.6 Sample preparation and LC-MS/MS analysis for proteomics analysis

Cells were lysed in denaturing urea lysis buffer (9 M urea, 0.1 M Tris HCl pH 8.5, 2 mM EDTA, 1× PhosStop (Roche), and phosphatase inhibitor cocktails 2 and 3 (Sigma), then sonicated. Lysates were processed and labelled with TMT-10plex reagents (ThermoFisher Scientific). Samples were then fractionated using a 4.6 mm × 250 mm Extend-C18 column (Agilent), on an Agilent 1200 Series HPLC. The fractions were pooled in a checkerboard manner, dried down, desalted by an Empore C18 stage tip, and re-suspended in 0.1% formic acid.

### 2.7 LC-MS/MS analysis

LC-MS/MS analysis was performed using a QExactive HF mass spectrometer (ThermoFisher Scientific) coupled to an EASY-nLC 1000 system (ThermoFisher Scientific). Peptides were separated on a 75 μm by 50 cm EASY-Spray analytical column (Thermo Fisher Scientific) at 50 °C. The mass spectrometer was set to acquire in a data-dependent mode (Top10). Full scans were acquired at 60,000 resolution, with a target of 3 × 10^6^ ions with a maximum injection time of 20 ms. The top intense ions were fragmented by MS^2^ scans were fragmented by HCD (NCE 33%) and acquired at 60,000 resolution, with a target of 1 × 10^6^ ions and a maximum injection time of 60 ms. MaxQuant version 1.5.3.30 was used to process MS data. The false discovery rate (FDR) was set at <0.01, enzyme was set to trypsin, and missed cleavages was set at <2. The Human UniProt FASTA database (March 2015) was used for peptide identification, with cysteine carbamidomethylation as a fixed modification and *N*-acetylation and oxidation of methionine as variable modifications. TMT quantification was performed by MaxQuant (Max Planck Institute of Biochemistry), using correction factors supplied with TMT reagents, reporter mass tolerance set to 0.01 Da, and a parent ion fraction (PIF) filter at 0.75.

### 2.8 Bioinformatics analysis of proteomics data

Protein lists exported from MaxQuant software were used for statistical analysis. The data normalization, principal component analysis (PCA), and statistical analysis of proteomics data were performed using R programming language (version 3.4.0, www.r-project.org). The raw data were normalised to the median intensity of all proteins within the TMT 10-plex set. The normalised intensity for each protein was then transformed to a relative ratio by dividing by the mean normalised intensity of the protein across the TMT 10-plex set. The LIMMA package (Ritchie et al., 2015) was used for statistical analysis of differential abundance. The p-values were adjusted for multiple comparisons using the Benjamini and Hochberg method (Benjamini et al., 1995); a protein was considered significantly different between groups if it had a FDR-adjusted p-value < 0.05.

### 2.9 RNA library preparation for RNA sequencing

RNA extraction was conducted using the RNeasy Micro kit (QIAgen). All RNA used for analysis conformed to a RIN of 8.8 or higher. 500 ng of high quality RNA was used as starting for poly-A library construction. Automated poly-A library construction was completed using TruSeq Stranded mRNA sample Preparation kit (Illumina). Individual libraries were quality controlled for size distribution and concentration using a LabChip GX and KAPA library quantification kit (Kapa Biosystems). Pooled libraries were clustered on a cBot (Illumina) using HiSeq PE Cluster kit V4 (Illumina) and sequenced on a HiSeq 2500 Sequencing system (Illumina) using HiSeq SBS kit V4 (Illumina) with 50/50 paired end sequencing at a read depth of approximately 30 million fragments in high output mode. Sequencing run quality control parameters were uploaded to BaseSpace (Illumina) and reviewed. Completed runs were considered high quality if >85% of bases above Q30 and Cluster Pass Filter >85%.

### 2.10 Bioinformatics analysis of transcriptomics data

Sequencing reads were aligned to reference genome (hg38) using OSA aligner (OmicSoft Sequence Aligner (Hu et al., 2012). Quality control for the sequence alignment involved the analysis of sequence quality, GC content, and 5′-3′ gene body coverage. Aligned reads were then counted against gene model annotation (gencode v23) to obtain gene level expression values by using RSEM (Li and Dewey, 2011). DESeq2 (Love et al., 2014) was used for gene expression normalization. The regularised log transformation function in DESeq2 was used to transform the raw count data to the log2 scale, minimising differences between samples for rows with small counts (transcripts with low expression), and normalising to library size. These values were used to perform PCA analysis for biological QC and downstream differential analysis. DESeq2 generalized linear model (GLM) was used for differential analysis. Transcripts with >0.5 Fragments per Kilobase of transcript per Million mapped reads (FPKM) were considered to be robustly detected. Differentially expressed gene (DEG) signature was defined by using the following criteria: FPKM > 0.5, false-discovery-rate (FDR)-adjusted p-value < 0.05 and absolute fold change (FC) > 1.5.

### 2.11 Combined bioinformatics analysis of proteomics and transcriptomics data for dual omics analysis

Proteomics data was multiplied by 1.4 to compensate for TMT-10plex labelling compression. Transcriptomics and proteomics datasets from 35 and 56 DIV for iPSC-derived dopaminergic neuronal cultures were combined to produce integrated FC and adjusted p-values. Briefly, if a protein was significantly differentially expressed at 35 DIV, then 35 DIV protein data was used; if not, and the protein was significantly differentially expressed at 56 DIV, then 56 DIV protein data was used. If the protein was not significantly differentially expressed at either time point, and the gene was significantly differentially expressed at 35 DIV, then 35 DIV gene data was used. If this also was not significant, but significantly different gene expression was seen at 56 DIV, then the 56 DIV gene data was used. Enrichment analyses of gene ontology (GO) terms and KEGG pathways were performed on this integrated dataset by applying Ensemble of Gene Set Enrichment Analyses (EGSEA) (Alhamdoosh et al., 2016) and utilizing all differentially expressed proteins/genes with p < 0.05 and a FC > 1.5 in either direction as input. Significant pathways with adjusted p-value < 0.05 were reported.

### 2.12 Live imaging

iPSC-derived dopaminergic neurons were grown until 48 DIV and were then imaged with FM1-43 (Thermo Fisher Scientific). Cells were washed in HBSS−/− supplemented with 5 mM glucose and 10 mM HEPES. 2 μM FM1-43 was then applied for 1 min before uptake was induced with 75 mM NaCl and 10 mM KCl and left for 1 min before being washed in HBSS−/− solution (Thermo Fisher Scientific). Images were acquired at 37C in 5% CO_2_ on the Opera Phenix (Perkin Elmer) at 63× magnification. Images were quantified in ImageJ by measuring the fluorescence intensity of puncta at each timepoint.

### 2.13 Neurite regrowth assay

iPSC-dopaminergic neuron cultures were grown until 33 DIV in 96-well plates (Griener). A scratch was applied through the centre of each well, and medium replaced. Brightfield images were captured every 24 h for 7 days using the Phenix Opera (Perkin Elmer). Cell coverage of the well over time was measured using the Harmony software (Perkin Elmer).

**Table:** Primary antibodies.

Antibody	Product code	Manufacturer	Application
FOXA2	AF2400	R&D systems	ICC
TH	sc-25269	Santa Cruz	ICC
TH	Chemicon	AB152	EM
TUJ1	ab107216	Abcam	ICC
β-actin (HRP-conjugated)	ab49900	Abcam	Western
Dynamin-1	ab52611	Abcam	Western
Endophilin I-III	sc-30101	Santa Cruz	Western/IF/ICC
Clathrin Heavy Chain	ab21679	Abcam	Western/IF/ICC
LRRK2	73-253	Antibodies Incorporated	Western
RAB5B	ab72907	Abcam	Western
RAB7	ab137029	Abcam	Western
RAB10	MABN730	Millipore	Western
Caveolin-1	3267	Cell Signalling	Western
Synapsin I and II	106004	Synaptic systems	Western
Synapsin (Phospho Ser603)	PA1-4604	Invitrogen	Western
Rab11	ab3612	Abcam	Western
Rab3a	ab3335	Abcam	Western

### 2.14 Western blotting

Samples were homogenised in RIPA buffer containing complete protease inhibitor cocktail (Roche) and phosphatase inhibitors (Sigma Aldrich) and a BCA assay was used to determine concentration. Samples were further diluted, Laemmli buffer added and boiled for 5 min. Samples were loaded and ran on 4–15% Criterion-TGX gradient gels (BioRad) and transferred to PDVF membranes. Membranes were blocked in 4% milk for one hour at room temperature followed by primary antibody incubation at +4 °C overnight. Membranes were then developed using immobilon western chemiluminescent HRP substrate (Millipore) and visualised on a ChemiDoc (BioRad).

### 2.15 Immunofluorescence

Rats were terminally anesthetised and transcardially perfused and iPSC derived neurons fixed in 4% paraformaldehyde solution (Sigma). Brains were dehydrated through an ethanol gradient prior to being paraffin-embedded, sectioned to 8 μm thick sections and dewaxed preceding citrate antigen retrieval and blocking. Following this brain sections were washed and then incubated with the appropriate primary and secondary antibodies (see table above). Nuclei were stained with DAPI and sections were mounted with fluorsave (Millipore). All analysis was done blind. Clathrin and endophilin puncta analysis was carried out in ImageJ by creation of a macro that first converted images to 8-bit prior to thresholding and particle analysis was set at 2–50 pixels.

### 2.16 Electron microscopy

Electron microscopy (EM) was performed as previously described in (Janezic et al., 2013). Briefly, rats were terminally anesthetized and transcardially perfused using 4% PFA and 0.1% glutaraldehyde. Coronal free floating sections were cut to 60 μm using a vibratome and then incubated with tyrosine hydroxylase primary antibody (Chemicon). Dopaminergic terminals were revealed by silver-intensified immunogold conjugated secondary antibodies (Nanoprobes) and samples were dehydrated and embedded in Durcapan ACM resin (Fluka). Serial sections of dorsal striatum ~50 nm were cut and collected onto copper grids. Prior to examination under the electron microscope grids were lead stained and samples were blinded. Terminals containing five or more immunogold particles were identified and imaged at 12,000×. A total of 50 terminals across two grids were imaged per rat, achieving 150 terminals per genotype. EM image analysis was carried out using the PointDensity and PointDensitySyn plugins in ImageJ (Anwar et al., 2011; Larsson and Broman, 2005). TH-positive profiles were identified as containing five or more immunogold particles and the perimeter delineated. The centre of each synaptic vesicle was labelled and was counted, provided that 50% or more of the membrane was visible.

### 2.17 Animal procedures

All animal procedures were carried out under the United Kingdom Animals (Scientific Procedures) Act (1986). Previously described *LRRK2*-expressing BAC transgenic rats were housed with littermate controls (Sloan et al., 2016). Rats were housed in a 12-h light-dark cycle with ad-libitum access to food and water. Both sexes were used throughout this study.

### 2.18 Human tissue

Paraffin embedded post-mortem tissue from *LRRK2-G2019S* Parkinson's cases and age matched controls were supplied by the Queens Square brain bank as 5 μm sections.

### 2.19 Statistics

Graphpad 7 software was used for statistical analysis. All data were analysed for statistical significance using unpaired *t*-test, one-way ANOVA or two-way ANOVA. All data are presented as means ± SEM.

## 3 Results

### 3.1 Control and *LRRK2* iPSC lines produce ventral midbrain dopaminergic neurons

**Fig. 1:**
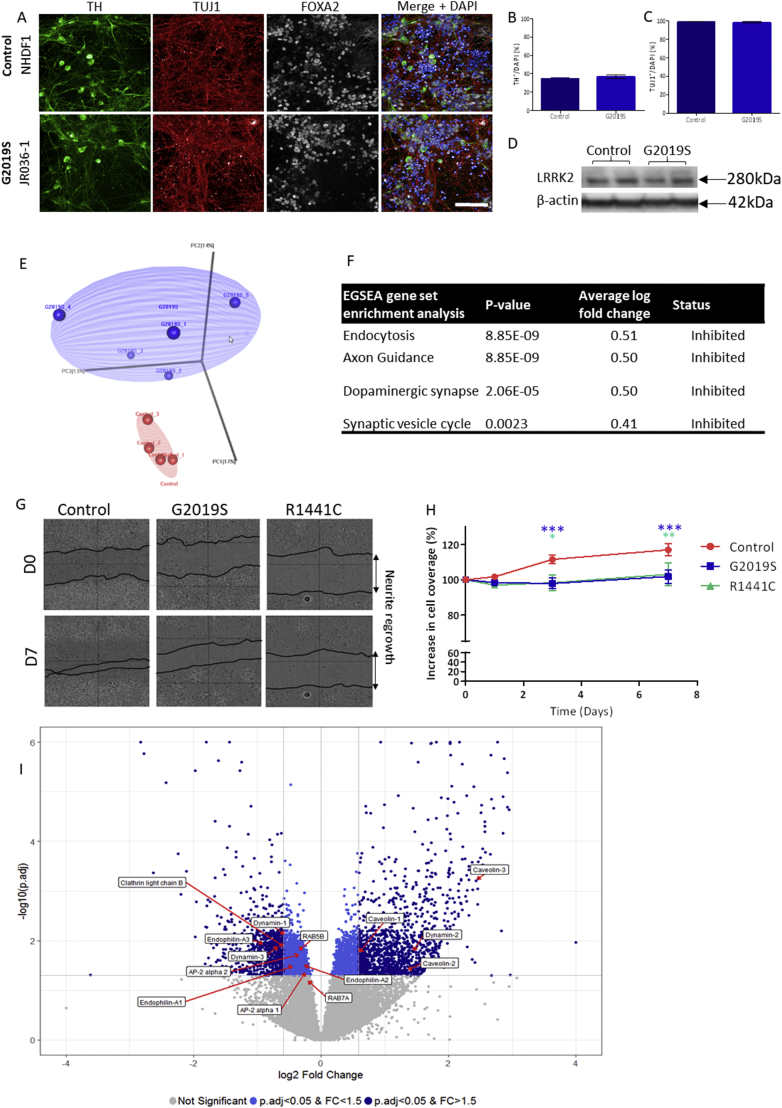
Integrated transcriptomic and proteomic analysis reveals the endocytic pathway to be the most dysregulated pathway in iPSC derived dopaminergic neurons from LRRK2-G2019S PD. (A) Representative images of iPSC-derived dopaminergic neurons showing staining for TH, Tuj1 and FOXA2 with the percentage of TH and TUJ1 positive cells demonstrated for both controls and LRRK2-G2019S in B and C. Scale bar 100 μm. (D) Representative western blots demonstrating LRRK2 expression in 35 DIV iPSC-derived dopaminergic cultures. Unpaired *t*-test. (E) PCA plot of integrated transcriptomic and proteomic data demonstrating the separation of iPSC-derived dopaminergic cultures from five G2019S patients and four controls. (F) Integrated proteomics and transcriptomics reveal a number of significantly dysregulated pathways with endocytosis being the most significantly altered. Representative images of neurite regrowth at D0 and D7 of scratch assay (G, H) showing significantly reduced regrowth into the scratch area in both LRRK2-G2019S and LRRK2-R1441C lines compared to controls. *p ≤0.05, **p ≤0.01, ***p ≤0.001 2-way ANOVA. (I) Volcano plot demonstrating fold change and p-values in the combined transcriptomic and proteomics dataset, with key endocytic proteins highlighted.

iPSCs were derived from four healthy controls, and eight patients with PD who carry either the *LRRK2-G2019S* or *LRRK2-R1441C* mutation (Figs. S1, S2). Cells were differentiated to midbrain dopaminergic neurons as previously described until 35 DIV or 56 DIV (Kriks et al., 2011). The composition of the cultures was consistent across genotypes and multiple differentiations (Figs. 1A–C, S1); on average (mean ± SD) 32.7 ± 4.3% of cells expressed tyrosine hydroxylase (TH), 98.2 ± 1.3% expressed β-tubulin (TUJ1), and 83.7 ± 5.9% expressed forkhead box protein A2 (FOXA2). LRRK2 protein expression can be seen in both controls and *LRRK2-G2019S* lines (Fig. 1D).

### 3.2 Integrated transcriptomic and proteomic analysis of *LRRK2-G2019S* iPSC-derived dopaminergic cultures reveals dysregulation of endocytosis and axon guidance

To understand the effects of the *LRRK2-G2019S* mutation on the transcriptome and proteome of iPSC-derived dopaminergic cultures, control and *LRRK2-G2019S* iPSC-derived dopaminergic neurons were analysed using RNA-seq and mass spectrometry. To investigate any effect of neuronal maturity, cells were analysed at both 35 and 56 DIV.

Transcriptomic analysis revealed that a total of 16,893 genes were detected in samples harvested at 35 DIV and 18,146 genes were detected in samples harvested at 56 DIV. Principal component analysis (PCA) was conducted for these data and demonstrated clear separation of the two genotypes at each timepoint (Fig. S3A, B). Further investigation revealed that at 35 and 56 DIV, 2238 and 2572 genes, respectively, were differentially expressed between genotypes (false-discovery rate (FDR) adjusted p < 0.05; fold change (FC) > 1.5).

Proteomic analysis was conducted using LC-MS/MS. To enable multiplex analysis, samples were labelled using TMT-10plex tags to create TMT sets for each timepoint. A total of 10,502 proteins were detected in samples harvested at 35 DIV, and 10,501 proteins were detected in samples harvested at 56 DIV. Similar to the transcriptomic analysis, PCA of these data demonstrated a clear separation between genotypes at each timepoint (Fig. S3C, D). Comparison of data from the two genotypes revealed that at 35 and 56 DIV, 2231 and 1439 proteins, respectively, were differentially expressed (FDR adjusted p < 0.05; FC > 1.5).

Given that data from lines expressing the *LRRK2-G2019S* mutation clustered separately from the controls at the protein and gene levels at both timepoints, the data were combined into a single dataset (the integrated omics dataset) for further analysis. PCA of the integrated dataset demonstrated clear separation of the *LRRK2-G2019S* and control groups (Fig. 1E). Interestingly, we noted that, although both controls and *LRRK2-G2019S* cells cluster separately by PCA analysis, the controls cluster more tightly in comparison to the *LRRK2-G2019S* cells possibly suggesting some heterogeneity in cellular effects of the *LRRK2-G2019S* mutation**.** Ensemble of Gene Set Enrichment Analyses (EGSEA) (Alhamdoosh et al., 2016) of the integrated dataset revealed endocytosis and axon guidance as the two most significantly perturbed pathways, both of which were predicted to be inhibited in the presence of the G2019S mutation (Fig. 1F).

The *LRRK2-G2019S* mutation has previously been demonstrated to disrupt axon guidance in iPSC-derived dopaminergic neurons (Borgs et al., 2016; Reinhardt et al., 2013; Sánchez-Danés et al., 2012; Su and Qi, 2013). We therefore conducted a neurite regrowth assay, in which a scratch was applied to the cultures and neurites would grow to fill the scratch area, to confirm this effect in our cultures, as well as in cultures from patients carrying the less common *LRRK2-R1441C* mutation (Fig. 1 G, H). Owing to the wealth of literature surrounding the effect of *LRRK2* mutations on neurite structure, we then focussed on understanding the impact of *LRRK2* mutations on endocytosis.

### 3.3 *LRRK2* mutations regulate expression of endocytic machinery in iPSC-derived dopaminergic cultures

Our integrated omics approach provided the power to identify a much more extensive array of altered endocytic genes and protein expression levels than previous studies have reported (Fig. S4). The magnitude and significance of the changes in key endocytic genes/proteins in the integrated omics dataset are highlighted in Fig. 1I (enlarged version Fig. S5). Of particular note, endophilin-III, which is essential for sensing the curvature in the region of membrane that is to be endocytosed, and dynamin-1, which acts to aid budding and scission of vesicles formed during CME, were significantly downregulated in the presence of the *LRRK2-G2019S* mutation (FC 0.54, p = 0.012; FC 0.68, p = 0.008; respectively). Additionally, clathrin light chain b, but not clathrin light chain a or heavy chains 1 or 2, was also downregulated in the *LRRK2-G2019S* lines (FC 0.67, p = 0.013).

We also detected significant changes in the expression levels of 25 RAB proteins, accounting for over a third of the 70 members of the RAB family (Fig. S4). These included RABs previously linked to LRRK2 biology; RAB5B (FC 0.78, p = 0.016), which localises to the early endosome; whereas RAB7, which localises to the late endosome and is important for its fusion with the lysosome, only showed a non-significant trend towards downregulation (FC 0.86, p = 0.06).

**Fig. 2:**
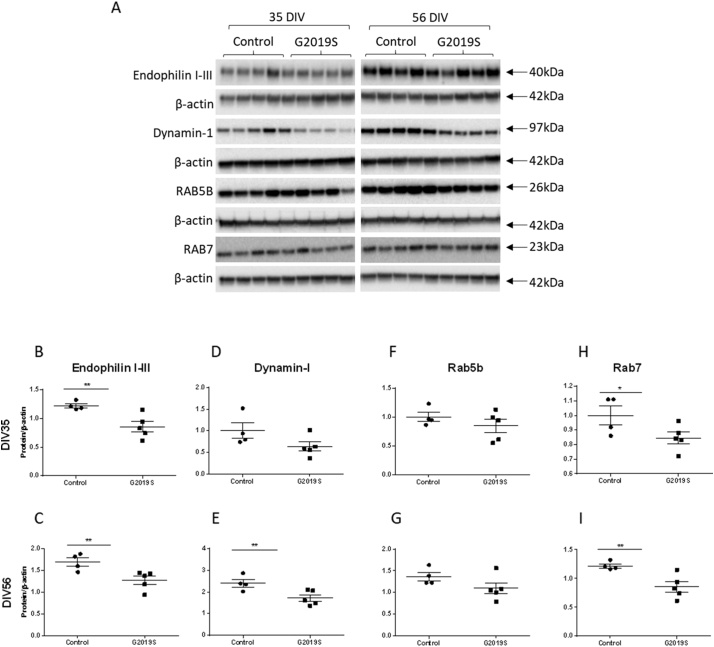
Endocytic protein expression in LRRK2-G2019S iPSC-derived dopaminergic cultures is reduced at DIV35 and 56. (A) Representative western blot images of iPSC-derived dopaminergic neuron samples at both DIV 35 and 56, with quantification for each protein normalised to β-actin (B–I). Graphs show mean ± SEM from four controls and five LRRK2-G2019S lines, from three independent differentiations. Significance was assessed using a *t*-test between controls and G2019S lines *p < 0.05, **p < 0.01.

Western blot analysis confirmed that the changes detected in the integrated omics dataset in *LRRK2-G2019S* neurons were robust (Fig. 2). In agreement with the integrated omics data, levels of the proteins endophilin I-III, were found to be significantly decreased in *LRRK2-G2019S* iPSC-derived dopaminergic cultures compared with controls at both DIV 35 and 56 (Fig. 2A–C). Dynamin-1 demonstrated a trend towards downregulation at DIV35 and reached significance at 56 DIV in *LRRK2-G2019S* cultures (Fig. 2A, D, E, Fig. S6). RAB5B and RAB7 were also downregulated (Fig. 2A, F–I, Fig. S6) with RAB7 reaching significance at both DIV35 and 56 in *LRRK2-G2019S* cultures compared to controls. Finally, we investigated whether the same effects were seen in iPSC-derived dopaminergic cultures carrying the *LRRK2-R1441C* mutation to probe the effect of GTPase domain mutations on the endocytic pathway (Fig. S6). At this point in the study only two *LRRK2-R1441C* iPSC lines were available, precluding statistical analysis, and the data are shown for indicative purposes only. However, in all cases the *LRRK2-R1441C* iPSC-derived dopaminergic cultures demonstrated a similar pattern of change for all four proteins consistent with the findings obtained for the *LRRK2-G2019S* mutation in the dual omics analysis and by western blotting.

### 3.4 Clathrin-mediated endocytosis is impaired in iPSC derived dopaminergic cultures carrying the *LRRK2-G2019S* or *LRRK2-R1441C* mutation

**Fig. 3:**
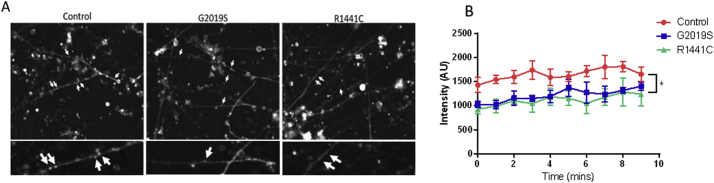
Endocytic function in LRRK2 iPSC-derived dopaminergic cultures is reduced. (A) Representative images of FM1-43 uptake across genotypes at DIV47 with quantification shown in (B). Images were taken over a period of 10 min and puncta were analysed from two separate wells per line, with 10 puncta being analysed over time per well. Graphs show mean fluorescent intensity ± SEM. *p ≤0.05 effect of genotype, 2-way ANOVA; n = 3–4 iPSC lines per genotype.

Given that three of the most critical proteins required for CME - clathrin, endophilin and dynamin - were downregulated in our *LRRK2*-PD iPSC-derived dopaminergic neurons, we predicted CME would be impaired in these cells. Following neuronal synaptic vesicle release, CME is initiated to recover and recycle synaptic vesicle components from the plasma membrane. Uptake of the lipophilic dye FM1-43 can be used to measure this process as described in materials and methods. iPSC-derived dopaminergic cultures were exposed to FM1-43 for a minute before the addition of potassium chloride to induce synaptic vesicle release. iPSC-derived dopaminergic cultures from *LRRK2-G2019S* and *LRRK2-R1441C* patients had significantly reduced uptake of the FM1-43 dye compared with control neurons, demonstrating a reduction in CME (Fig. 3).

### 3.5 Key endocytic protein levels are perturbed in aged rats carrying *LRRK2* mutations

To further understand the relevance of our findings in dopaminergic cultures we employed an in vivo model of PD. Striatal tissue from 22-month old BAC transgenic rats expressing the human *LRRK2* wild-type (*hWT*), *LRRK2-G2019S* or *LRRK2-R1441C* transgene (Sloan et al., 2016) were studied for changes in endocytic protein levels.

**Fig. 4:**
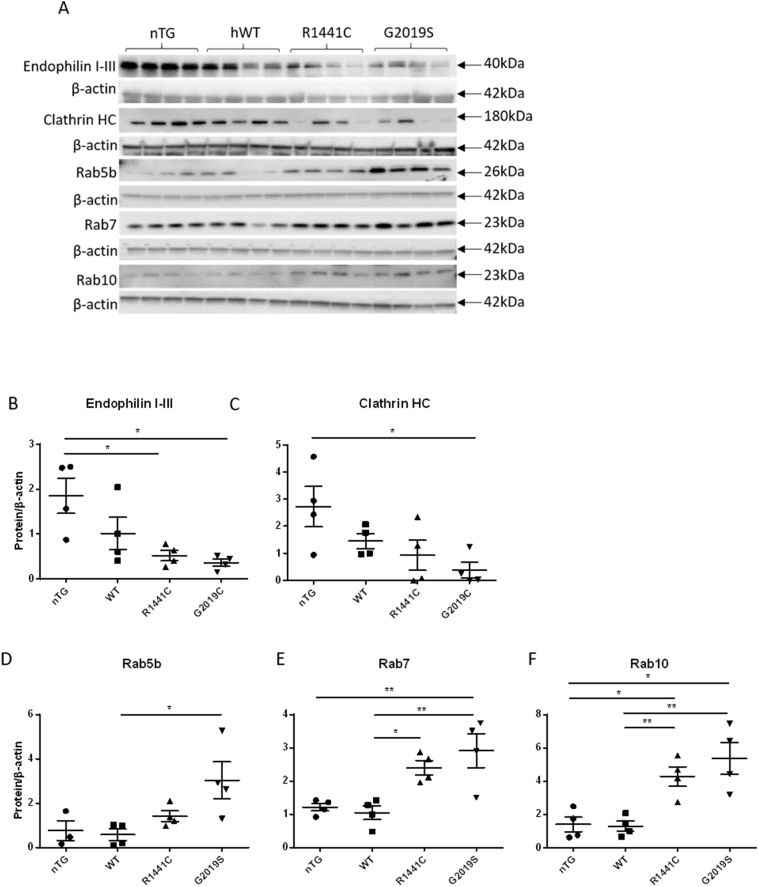
Perturbations of endocytic protein expression in aged LRRK2 BAC transgenic rats. (A) Representative western blot images of Endophilin, Clathrin and Rabs 5b, 7 and 10 from 22-month old rat striatal tissue from non-transgenic (nTG) rats and rats expressing human WT (hWT), human LRRK2-R1441C or human LRRK2-G2019S. (B–F) Quantification of western bots compared to β-actin. Data are expressed as optical density normalised to β-actin. Graphs show mean ± SEM, *p ≤0.05, **p ≤0.01 ANOVA, Tukey's post-hoc; *n* = 4 animals per genotype.

Terminals of dopaminergic neurons originating in the SNpc project to the dorsal striatum and, due to their high synaptic demand and arborisation, are highly dependent on effective endocytic recycling (Bolam and Pissadaki, 2012; López-Murcia et al., 2014). In agreement with our findings in iPSC-derived dopaminergic neurons, western blot analysis revealed significantly reduced levels of clathrin heavy chain and endophilin I-III in rats expressing *LRRK2* mutations compared with non-transgenic (nTG) controls; however, levels were not significantly different from rats expressing *hWT-LRRK2* (Fig. 4A–C). Conversely, RAB5B, RAB7 and RAB10 were upregulated in *LRRK2-G2019S* and *LRRK2-R1441C* rats compared with those expressing *hWT-LRRK2* (Fig. 4A, D–F). RAB3A and RAB11, which are involved in synaptic vesicle exocytosis and the recycling endosome, respectively, and were downregulated by the *LRRK2-G2019S* mutation in the integrated omics analysis, were unaltered (Fig. S7). Dynamin-1 and Caveolin-1 were also unaltered in 22-month old *LRRK2* BAC rat striatal tissue (Fig. S7). This endocytic phenotype was only present in aged rats; no changes in levels of these endocytic proteins were seen in 12-month old rats (Fig. S8).

**Fig. 5:**
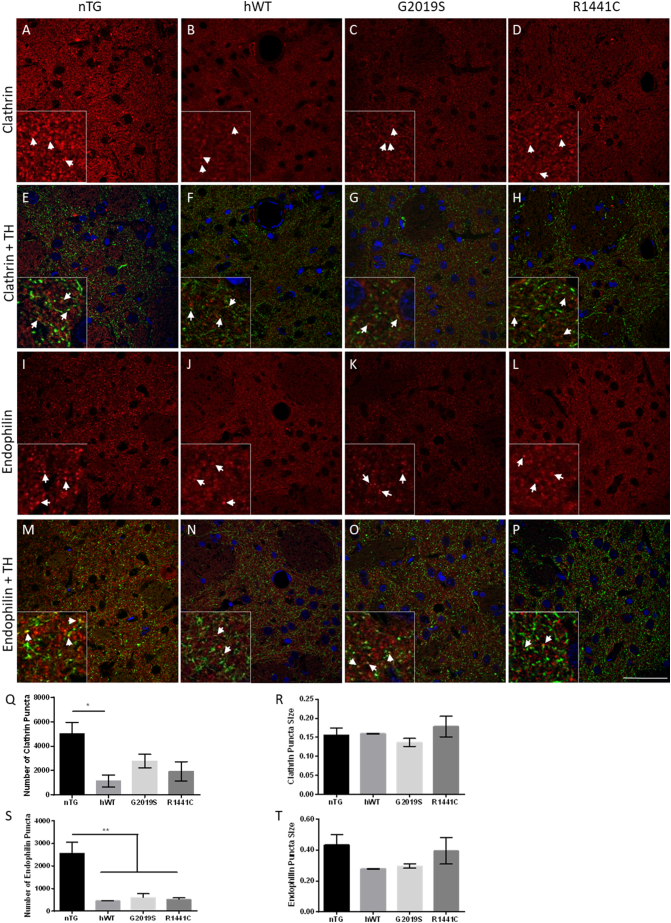
Transgenic LRRK2 rats have significantly reduced numbers of endophilin and clathrin puncta. (A–P) Representative images of 22-month old rat striatal sections across genotypes of clathrin and endophilin I-III staining with the addition of TH (green) in the merged images. Insets show zoomed in regions showing puncta in more detail. White arrowheads identify puncta and puncta co-localising with TH in merged images, scale bar represents 100 μm. Quantification of clathrin puncta number (Q) and size (R) as well as endophilin puncta number (S) and size (T). Graphs show mean ± SEM, *p ≤0.05, **p ≤0.01 ANOVA, Tukey's post-hoc; *n* = 3 animals per genotype.

To further investigate changes in endophilin and clathrin protein levels, dorsal striatal sections of aged rats were immunostained for endophilin I-III and clathrin heavy chain and the number of puncta quantified for each. The total number of puncta for each protein was significantly reduced in *hWT-LRRK2* expressing rats whilst showing a clear reduction in *LRRK2-G2019S* and *LRRK2-R1441C* rats compared with nTG rats, and the number of endophilin puncta were also reduced with the *LRRK2-G2019S* and *LRRK2-R1441C* mutations. In each case, the average size of these puncta was unchanged (Fig. 5). Interestingly, these data demonstrate that overexpression of *hWT-LRRK2* has an impact on the levels of these proteins suggesting a fundamental role of LRRK2 in endocytosis.

### 3.6 Mutations in *LRRK2* lead to changes in synaptic vesicle dispersal

**Fig. 6:**
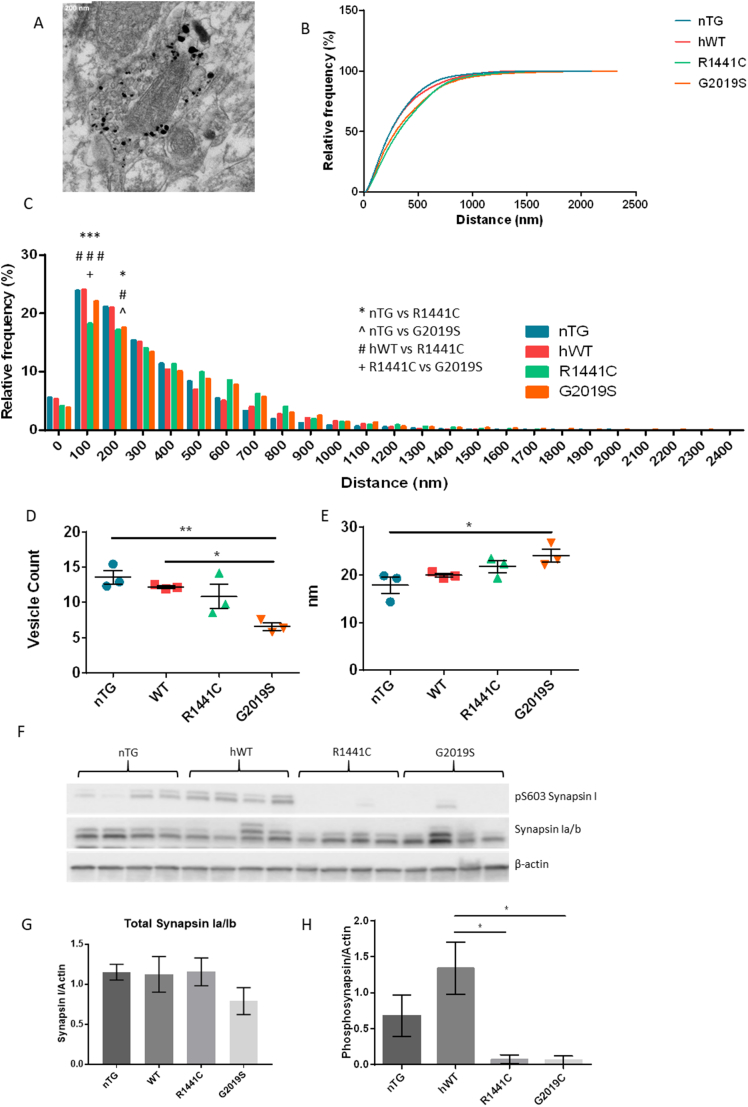
Synaptic vesicle distribution is altered in aged rats expressing LRRK2-G2019S and LRRK2-R1441C. (A) Immunogold labelled dopaminergic terminal, scale bar represents 200 nm. (B) Histogram showing the distribution of synaptic vesicles in dopaminergic terminals of the dorsal striatum of 22-month old LRRK2 BAC transgenic rats. (C) Histogram showing frequency of synaptic vesicles at different distances. 50 dopaminergic terminals assessed per animal; *n* = 3 per genotype. * nTG vs R1441C, ^ nTG vs G2019S; # hWT vs R1441C, + R1441C vs G2019S, *p ≤0.05, **p ≤0.01, ***p ≤0.001 2-way ANOVA, Tukey's post-hoc. (D) Average number of synaptic vesicles per dopaminergic terminal and the average synaptic vesicle diameter (E). 50 dopaminergic terminals assessed per animal; n = 3 per genotype. *p ≤0.05, **p ≤0.01, ANOVA, Tukey's post-hoc. Representative image and quantification of synapsin and phosphosynapsin (F, G, H) western blots in striatal tissue from 22-month old rats. Data are expressed as optical density normalised to β-actin. Graphs represent mean ± SEM, *p ≤0.05, **p ≤0.01 ANOVA, Tukey's post-hoc; *n* = 4 animals per genotype.

Previous work with these *LRRK2* BAC transgenic rat lines revealed dopaminergic signalling deficits including an age-related reduction in dorsal striatal dopamine release despite no alterations in total dopamine levels, and a motor impairment which is corrected with L-DOPA treatment (Sloan et al., 2016). Due to the apparent deficits we observe here in the endocytic pathway, which is crucial for recycling of synaptic vesicles (SVs), and the previously reported dopaminergic signalling phenotypes, we analysed dopaminergic profiles of the dorsal striatum using immunogold electron microscopy in 22-month old *LRRK2* BAC transgenic rats (Fig. 6A). There were no major morphological changes measured by profile area, perimeter or synapse length (Fig. S9). However, *LRRK2-G2019S* rats demonstrated both a significant reduction in the number of synaptic vesicles and synaptic vesicle diameter compared to controls (Fig. 6D–E). In both *LRRK2-G2019S* and LRRK2-R*1441C* rats there are significantly fewer synaptic vesicles close together and a greater number spaced further apart compared to controls indicating an altered distribution of synaptic vesicles (Fig. 6B–C).

In an attempt to understand this change in synaptic vesicle dispersion, we next investigated the levels of synapsin I, a protein key to tethering synaptic vesicles to each other and to actin filaments (Cesca et al., 2010). Although the total levels of synapsin I were unaltered across genotypes, levels of phospho-S603 synapsin I, which decides the proteins binding status to synaptic vesicles, were severely reduced in aged, but not young, rats expressing either the *LRRK2-R1441C* or *LRRK2-G2019S* mutations (Fig. 6D–F).

### 3.7 Clathrin and endophilin levels are increased in *LRRK2-G2019S* PD patient post mortem striatum

**Fig. 7:**
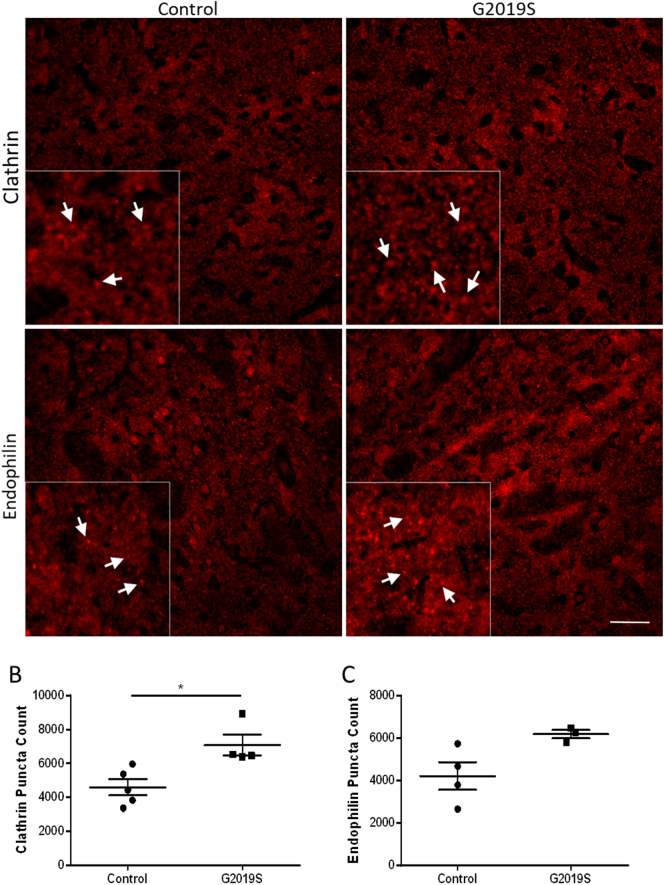
Human putamen post-mortem tissue from LRRK2-G2019S carriers reveals increases in the numbers of clathrin and endophilin puncta. (A) Representative images of clathrin and endophilin stained human post-mortem samples. Insets represent zoomed in regions showing puncta that are highlight using white arrows. Quantification of the number of both clathrin (B) and endophilin (C) puncta. Scale bar represents 50 μm; n = 3–5 per group. Graphs show mean ± SEM. *p ≤0.05 *t*-test.

To determine whether key proteins involved in the CME pathway were also dysregulated in *LRRK2* patients, we stained human post-mortem striatal tissue from PD patients carrying the *LRRK2-G2019S* mutation and age matched controls. There was an increase in the number of clathrin puncta and a trend towards an increase in the number of endophilin puncta in the putamen, but not the globus pallidus, of patient samples compared with controls (Figs. 7, S10). In PD patients, the putamen is heavily affected by disease pathology whereas the globus pallidus generally escapes degeneration (Hardman and Halliday, 1999a; Hardman and Halliday, 1999b; Jellinger and Attems, 2006). In both regions, as seen for the aged rats, there was no change in puncta size (Fig. S10). These findings suggest that the endocytic pathway is dysregulated in late stage disease.

## 4 Discussion

Our use of a novel integrated -omics analysis provided the power to demonstrate extensive dysregulation of the endocytic pathway by the *LRRK2-G2019S* mutation. This dysregulation is sufficient to lead to functional impairment of CME in *LRRK2-G2019S* and also in *LRRK2-R1441C* iPSC-derived dopaminergic cultures as demonstrated by reduced uptake of FM1-43. Furthermore, similar perturbations of this pathway were demonstrated in aged *LRRK2* BAC transgenic rats and finally in *LRRK2* PD post-mortem tissue. The importance of the endocytic pathway in PD pathology has been highlighted by previous work with *LRRK2* but also other disease causing mutations in genes such as *VPS35, DNAJC6, SYNJ1, GAK* and *Rab7L1* (Elsayed et al., 2016; Krebs et al., 2013; Nalls et al., 2014; Olgiati et al., 2016; Vilariño-Güell et al., 2011; Zimprich et al., 2011).

The unbiased integrated omics approach revealed axonal guidance and endocytosis as the two most perturbed pathways. We confirmed the presence of neurite regrowth phenotypes in our mutant *LRRK2* iPSC-derived dopaminergic neurons (Fig. 1), and then focussed on understanding the effect of *LRRK2* mutations on endocytosis. It should be noted that the endocytic pathway is crucial to the process of axonal outgrowth and so it is likely that impairment in endocytosis plays a role in the development of this phenotype (Tojima and Kamiguchi, 2015).

As neuronal activity results in the insertion of SVs into the membrane, these cells are highly reliant on efficient CME to retrieve SV proteins and maintain the plasma membrane. Therefore, changes in levels of key proteins related to this process are likely to have a detrimental impact on the cell. In both our iPSC-derived dopaminergic cultures and aged *LRRK2* BAC transgenic rats we demonstrated that the presence of *LRRK2* mutations significantly reduces levels of both clathrin and endophilin, two critical CME proteins. These reductions would be expected to impair normal CME, and this was demonstrated through the use of FM1-43 to be the case in iPSC-derived dopaminergic cultures from *LRRK2-R1441C* and *LRRK2-G2019S* PD patients, compared with cells from healthy controls. Reductions in clathrin levels by as little as 20% are also able to reduce synaptic transmission through reduction in the size of the ready releasable SV pool (RRP) and through reduction in quantal size (López-Murcia et al., 2014; Moskowitz et al., 2005). We observed a reduction in the total number of SVs in our *LRRK2-G2019S* rats compared with controls; however, it should be noted that true vesicle pools are hard to define in dopaminergic neurons and that previous work demonstrating a reduction in the size of the RRP have concentrated on other cell types in a neuronal *Lrrk2* knock-down model (Piccoli et al., 2011). We also demonstrated a change in the distribution of SVs in dopaminergic neurons in *LRRK2-R1441C* and *LRRK2-G2019S* rats compared to control rats, and evidence that tethering of vesicles is impaired in the presence of the *LRRK2-G2019S* and *LRRK2-R1441C* mutations. Furthermore, a previous study using these rat models demonstrated a reduction in dopamine release from the dorsal striatum using FCV despite no alterations in total dopamine concentrations being measured (Sloan et al., 2016). This may suggest a reduction in quantal size due to reduced levels of CME.

As previously discussed, endophilin levels were also significantly reduced in iPSC-derived dopaminergic neurons and in aged rats carrying *LRRK2* mutations, compared to the respective controls. These results corroborate the recent findings of Nguyen & Krainc who saw similar reductions in endophilin levels in iPSC from patients carrying the *LRRK2-R1441G* mutation (Nguyen and Krainc, 2018). Previously LRRK2 has been shown to phosphorylate endophilin, altering its membrane association, with both hyper- and hypophosphorylation leading to impairments in endocytosis (Matta et al., 2012). In our *LRRK2* mutant iPSC-derived dopaminergic neurons, we also saw a reduction in dynamin 1 levels, which suggests that scission of newly formed clathrin coated pits may be inhibited in these cells. Our integrated -omics analysis also revealed significant changes in numerous other endocytic proteins including the clathrin adapter protein AP-2, early endosome markers and the caveolin proteins. Interestingly, all members of the caveolin family were upregulated in this analysis; these are involved in clathrin-independent endocytosis and may compensate for the apparent deficit in CME demonstrated here (Parton and del Pozo, 2013). It has been previously demonstrated that LRRK2 is able to localise to caveolae, where caveolin proteins are localized, suggesting that LRRK2 may also have a function in this part of the endocytic pathway (Alegre-Abarrategui et al., 2009). Other highly upregulated proteins in our analysis included CHMP4C and PSD4 (also known as EFA6) which are required for the ESCRT-III complex and clathrin independent membrane recycling and remodelling respectively again suggesting that in the absence of efficient clathrin mediated endocytosis other mechanisms of membrane and receptor recycling are upregulated. CHMP4C is a crucial component of the ESCRT-III complex which is required for efficient multivesicular body sorting and formation (Schmidt and Teis, 2012). The ESCRT-III complex has previously been implicated in PD for its role in the transport of α-synuclein for degradation and shown that disruption to the complex leads to increased α-synuclein exocytosis (Spencer et al., 2016). Previous work has demonstrated that the PSD4 acts as an exchange factor for Arf6 which is an important mediator of endocytic processes (D'Souza-Schorey and Chavrier, 2006). Interestingly PSD4 is known to be able recruit endophilin to the flat areas of the plasma membrane and is considered to regulate the recycling of certain membrane receptors (Boulakirba et al., 2014; Casanova, 2007).

Despite decreases in endophilin and clathrin protein levels in the presence of *LRRK2* mutations in our in vitro and in vivo models, increases in both of these proteins were seen in post-mortem tissue from PD patients carrying the *LRRK2-G2019S* mutation compared with controls. Post-mortem tissue is representative of the very late stages of disease when most dopaminergic input to the striatum has been lost. Neither our in vitro or in vivo models recapitulate this loss. It is also possible that other neuronal cells types are compensating for the loss of dopaminergic innervation and so undergoing more synaptic activity. Nevertheless, our findings demonstrate a clear perturbation in the endocytic machinery in the brains of *LRRK2-G2019S* PD patients.

Strikingly, levels of 25 of the approximately 70-member family of RAB proteins were found to be significantly altered in the presence of the *LRRK2-G2019S* mutation in our integrated omics analysis. RABs are small proteins critical for intracellular vesicle trafficking and function within the endocytic pathway, and neuronal-specific RABs have been shown to have a predominant function at the synapse (Chan et al., 2011; Ridvan Kiral et al., 2018). We confirmed dysregulation of RAB5B, RAB7A and RAB10, which are localized to the early and maturing endosome, across our iPSC-derived dopaminergic cultures and aged rat models. Levels of these proteins were reduced in mutant iPSC-derived dopaminergic neurons and increased in striatal tissue of mutant *LRRK2* BAC transgenic rats, versus the relevant controls. This difference may be explained by the difference in disease stage represented by these two models. The iPSC-derived dopaminergic neurons likely represent a very early stage of PD pathogenesis, whereas the aged BAC transgenic rats represent an intermediate phase. In this context, our findings suggest that LRRK2 has an important role in the regulation of RAB protein levels and that LRRK2 mutations may have a biphasic effect over the time-course of PD pathogenesis. Providing further support to this hypothesis, levels of total RAB5 and RAB7 have also been shown to be increased in post-mortem brains of sporadic Alzheimer's disease patients compared with controls (Cataldo et al., 2000; Ginsberg et al., 2011; Ginsberg et al., 2010). This may be due to an over activation of the endocytic system or a blockage further up the *endo*-lysosomal pathway. A subset of Rab proteins have recently been identified as LRRK2 kinase substrates (Jeong et al., 2018; Liu et al., 2018; Steger et al., 2016) and previous work has identified interactions between LRRK2 and certain Rab proteins (Dodson et al., 2012; MacLeod et al., 2013; Shin et al., 2008). Interestingly, a recent study from Di Maio et al. has shown that WT LRRK2 kinase activity is upregulated in post-mortem nigral tissue from sporadic PD patients and that this is associated with increased RAB10 phosphorylation (Di Maio et al., 2018; Fan et al., 2018). Our results taken together with recent literature in the field highlight the importance of LRRK2 in the regulation of RAB proteins.

Given that alterations in the endocytic system can impact on the organisation and number of SVs, we investigated dopaminergic terminals of the dorsal striatum in 22-month-old transgenic rats. In the *LRRK2-G2019S* rats we observed significantly fewer synaptic vesicles which were significantly larger. This suggests a compensation by those synaptic vesicle remaining as previous work has demonstrated no loss of striatal dopamine in these rats (Sloan et al., 2016). We identified changes in the spatial dispersal of SVs in the presence of the *LRRK2-R1441C* or *LRRK2-G2019S* mutation compared with controls. This may be explained by the observed clear lack of synapsin phosphorylation at S603 present in our *LRRK2-G2019S* and *LRRK2-R1441C* rats which has also been previously demonstrated by others in *Lrrk2-G2019S* knockin mice (Beccano-Kelly et al., 2015). Synapsin orchestrates the tethering of SVs to each other and to actin filaments and, when phosphorylated at S603, reduces its binding, thus priming vesicles for release (Cesca et al., 2010; Fornasiero et al., 2012). In the almost complete absence of phosphorylation of synapsin in both aged *LRRK2-R1441C* and *LRRK2-G2019S* rats SVs are likely less primed for release which may explain the previously published reduced dopamine release measured by FCV in these models (Sloan et al., 2016).

## 5 Conclusions

We have used an integrated transcriptomics and proteomics approach to reveal the extensive dysregulation of the endocytic pathway caused by mutations in *LRRK2*. Together, our findings across PD *LRRK2* models and post-mortem brain tissue from PD patients carrying *LRRK2* mutations demonstrate that *LRRK2* mutations lead to clear and substantial changes in the endocytic pathway. Our findings also suggest that wild-type LRRK2 has an important role in regulating normal endocytic function. Further studies will be required to interrogate the more intricate role of LRRK2 in this pathway.

## Ethics approval

All animal procedures were carried out under the United Kingdom Animals (Scientific Procedures) Act (1986).

Human tissue was used in accordance with the local research ethics committee.

## Consent for publication

Not applicable.

## Availability of data and material

The datasets used during the current study are available from the corresponding author on reasonable request.

## Competing interests

Authors declare no conflict of interest.

## Funding

The work was supported by the Monument Trust Discovery Award from Parkinson's UK. HB was supported by an MRC Industrial CASE studentship. Samples and associated clinical data were supplied by the Oxford Parkinson's Disease Centre study, funded by the Monument Trust Discovery Award from Parkinson's UK, a charity registered in England and Wales (2581970) and in Scotland (SC037554), with the support of the National Institute for Health Research (NIHR) Oxford Biomedical Research Centre based at Oxford University Hospitals NHS Trust and University of Oxford, and the NIHR Comprehensive Local Research Network. The James Martin Stem Cell Facility, University of Oxford is financially supported by the Wellcome Trust WTISSF121302, the Oxford Martin School LC0910-004, the MRC Dementias Platform UK Stem Cell Network Capital Equipment and Partnership Awards (S.A.C.). The work was supported by the Innovative Medicines Initiative Joint Undertaking under grant agreement number 115439, resources of which are composed of financial contribution from the European Union's Seventh Framework Programme (FP7/2007e2013) and EFPIA companies' in kind contribution. The work of N.M.D. was funded by the Wellcome Trust (Investigator Award 101821) and the Medical Research Council of the United Kingdom (award MC_UU_12024/2). C.K. is the recipient of a career development award from the Hermann and Lilly Schilling Foundation and receives funding by the DFG (FOR2488; P1).

## Author contributions

N.C-R, H.B, S.A.C., J.G.M, B.G, K.L, W.H and R.W-M designed research; N.C-R, H.B, J.G.M, B.G, K.L, J.V, C.B, L.K, P.J, S. A. C performed experiments; N.C-R, H.B, J.G.M, B.G, K.L, J.V, C.B, L.K, P.J, S. A. C analysed data and N.C-R, H.B and R.W-M wrote manuscript.

All authors read and approved the final manuscript.

## Appendix A Supplementary data

Supplemental figures. Image 1
