# Supplementary material for: An integrated transcriptomics and proteomics analysis reveals functional endocytic dysregulation caused by mutations in LRRK2
Source: Neurobiol Dis. 2019 Jul;127:512–26. doi: 10.1016/j.nbd.2019.04.005 (PMC6597903; doi:10.1016/j.nbd.2019.04.005)
Supplement: Supplementary file 1 — Supplemental figures. [file mmc1.docx]

**Supplemental figure Legends**

**
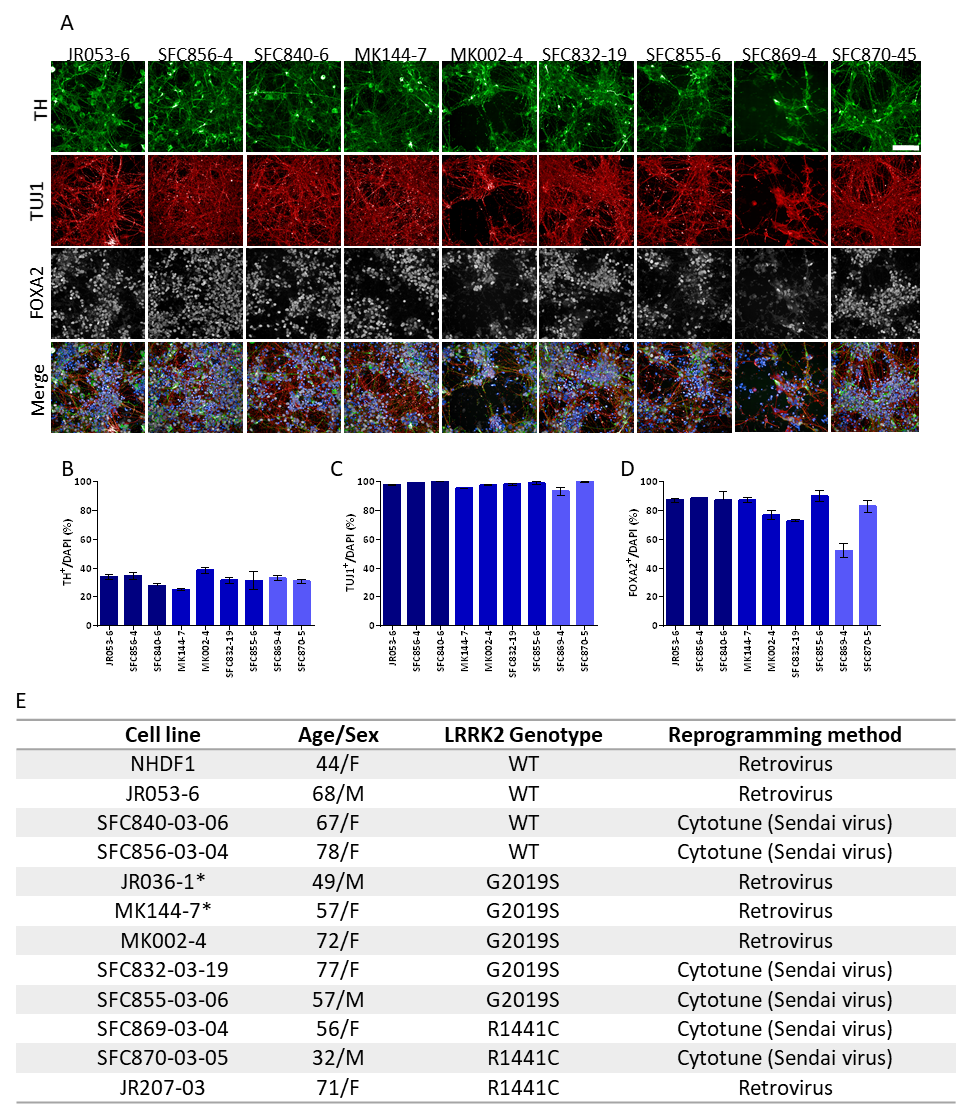
**

**Supplemental Figure S1**

**Characterisation of iPSC-derived dopaminergic neurons. (A)** Representative images of all iPSC-derived dopaminergic neurons used throughout this study stained for TH, TUJ1, FOXA2 and DAPI. Scale bar represents 100 µm. Quantification of the percentage of cells positive for TH **(B)**, TUJ1 **(C)** and FOXA2 **(D)** are shown. Graphs show mean ± SEM across multiple differentiations. One-way ANOVA. Details of all lines used are shown in **(E)** including reprogramming method. Siblings are denoted with an asterisk.


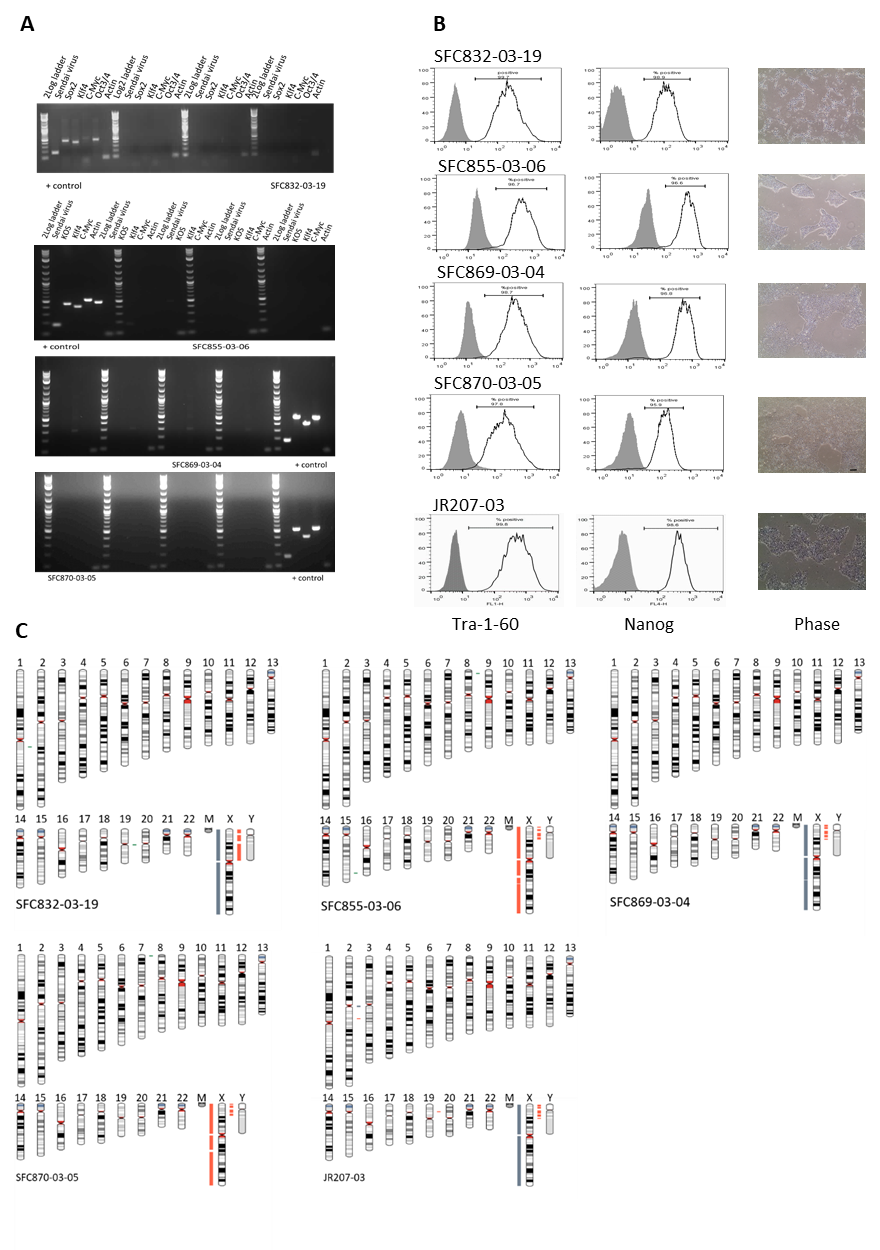


**Supplemental Figure S2**

**Reprogramming and characterization of previously uncharacterised iPSC lines from *LRRK2* G2019S and R1441C patients**

**(A)** Confirmation of clearance of Sendai virus reprogramming factors, with RT-PCR analysis for each transgene-containing virus. L, Log2 ladder; Se, Sendai backbone 181 bp; S, Sox2 451 bp; K, Klf4 410 bp; M, c-myc 532 bp; O, Oct-4 483 bp; KOS, 528bp; A, β-actin control 92 bp; + positive control fibroblasts infected with Cytotune 5 days previously. iPSC lines show the correct size band for β-actin, and no bands corresponding to the reprogramming virus PCR product sizes. **(B)** FACS analysis confirmation of expression of pluripotency markers Tra-1-60 and Nanog in iPSCs; open black plot represents antibody, filled grey plot represents isotype control; right-hand panel shows the expected iPSC colony morphology of cells 4 days post thaw (1 day post-thaw for SFC832-03-19), with high nucleus to cytoplasm ratio by phase-contrast microscopy. Scale bar = 100 µm. **(C)** Genome integrity assessed by Illumina Human CytoSNP-12v2.1 or OmniExpress24 SNP array and karyograms produced using KaryoStudio software (Illumina). Amplifications (green), deletions (orange) and LOH regions (grey) are shown alongside the relevant chromosome (except that in females the X chromosomes are annotated with grey, and single-copy sex chromosomes are annotated orange).


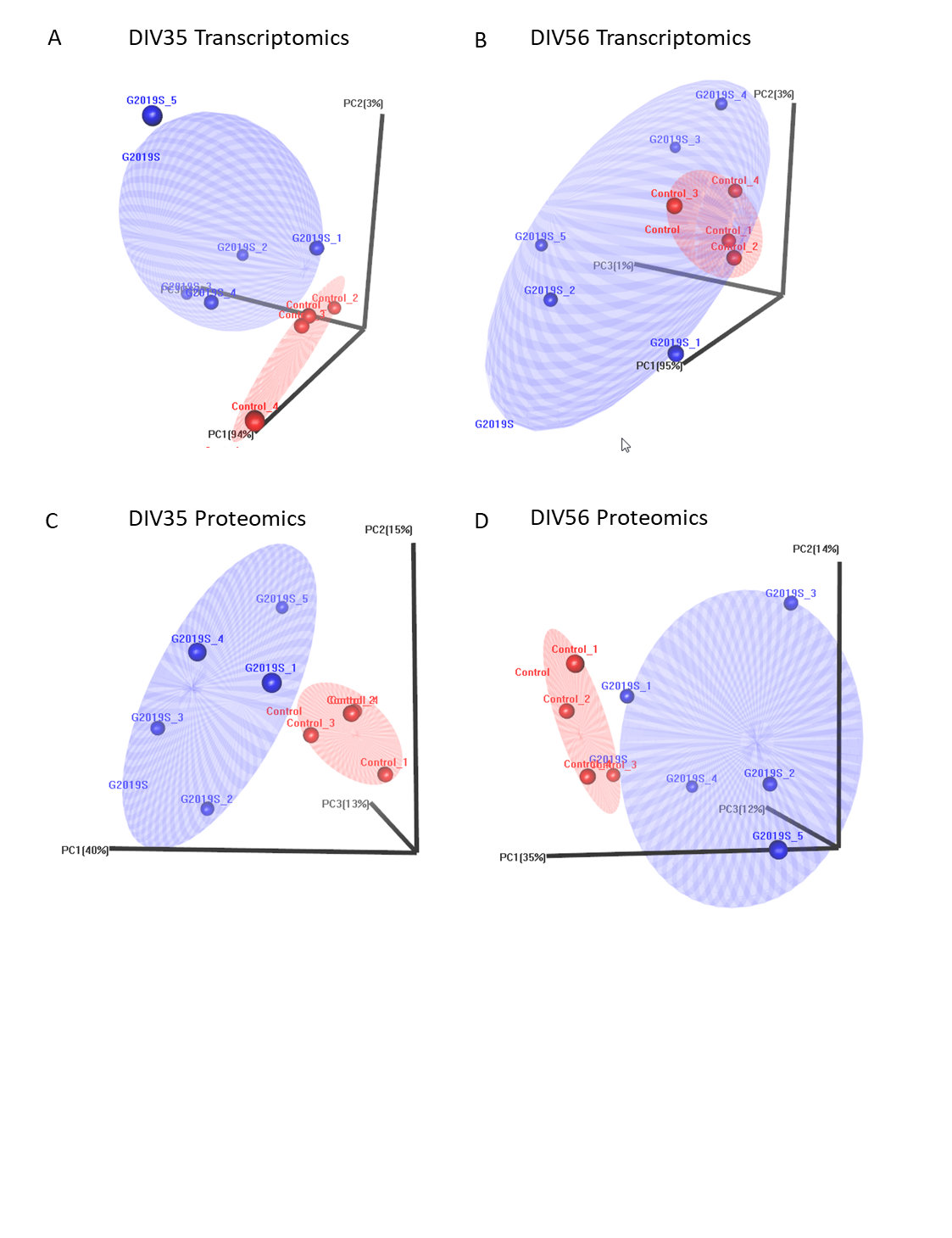


**Supplemental Figure S3**

**Principal component analysis of proteomic and transcriptomic data from iPSC-derived dopaminergic neurons prior to integration of datasets.**

Comparison of iPSC-derived dopaminergic neurons from G2019S LRRK2 patients and controls shows separation by PCA in transcriptomic data at both DIV35 **(A)** and DIV56 **(B)**. This is also the case for proteomic data at both DIV35 **(C)** and DIV56 **(D)**.


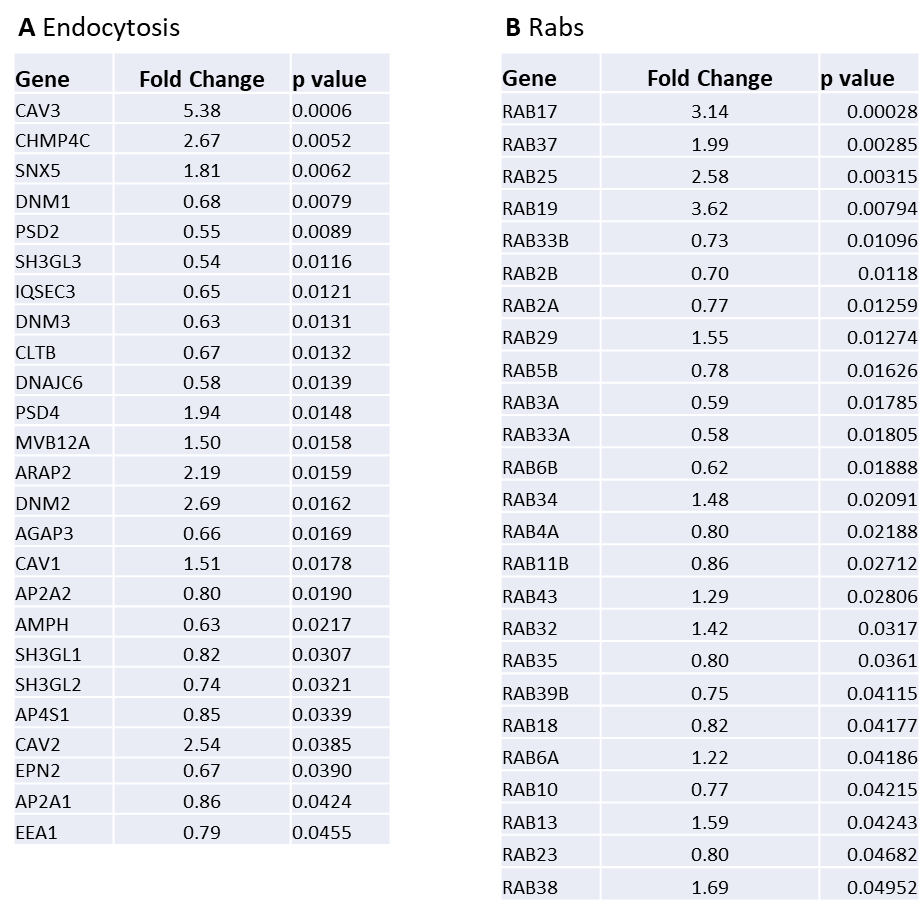


**Supplemental figure S4**

**Additional endocytic and Rab hits from the integrated omics approach.**

Tables showing additional endocytic **(A)** and Rab **(B)** hits from the integrated omics approach showing gene name, fold change and p-value.


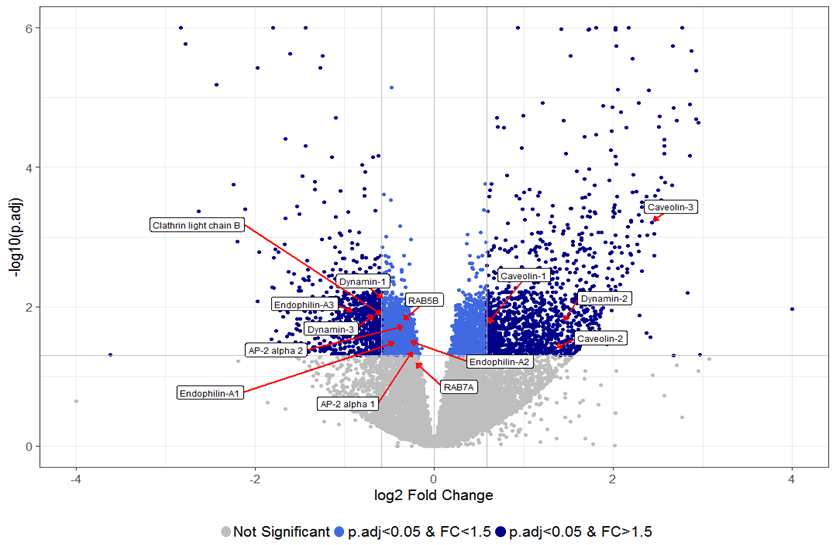


**Supplemental Figure S5**

Enlarged Volcano plot demonstrating fold change and p-values in the combined transcriptomic and proteomics dataset, with key endocytic proteins highlighted.

**
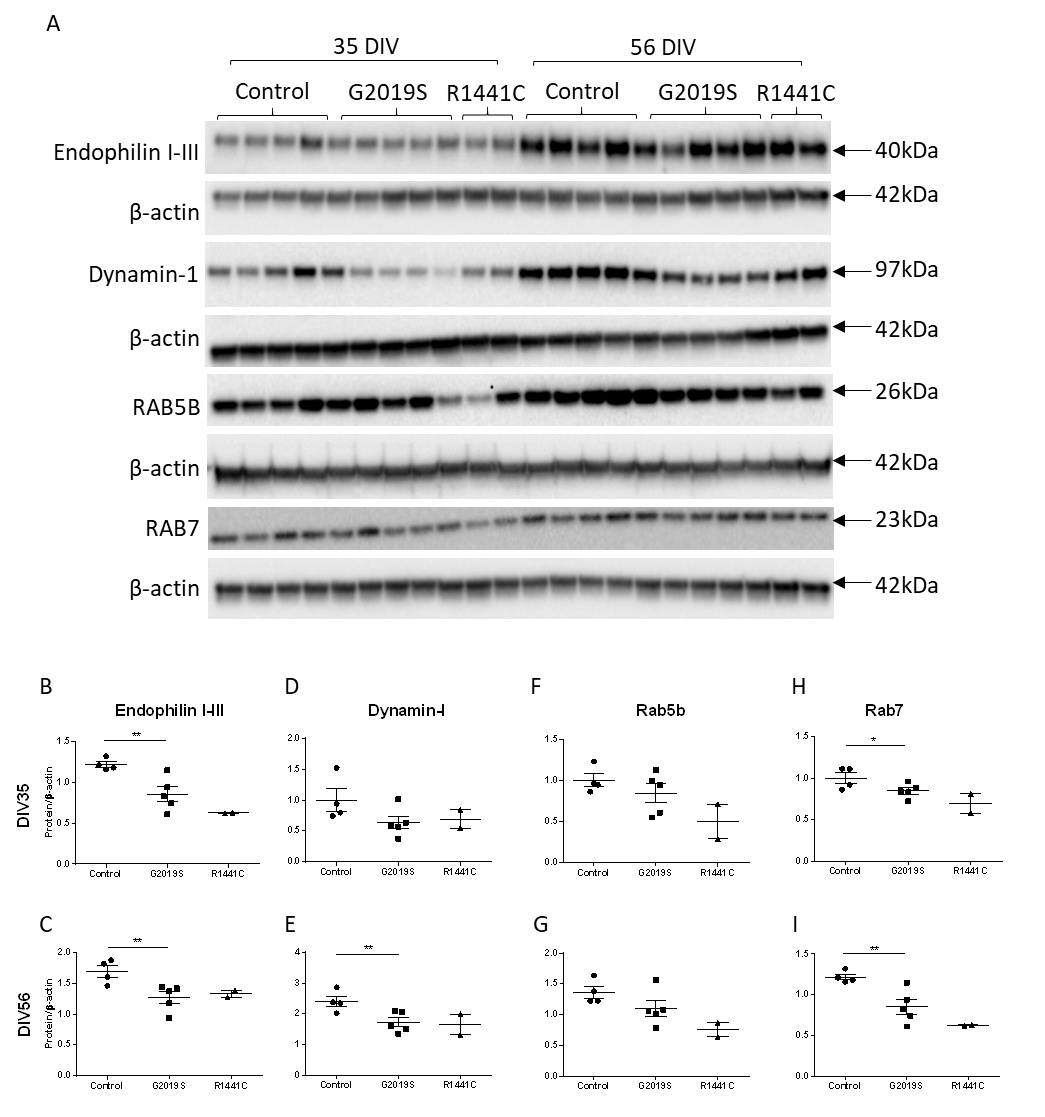
Supplemental Figure S6**

**Endocytic protein expression in LRRK2 iPSC-derived dopaminergic cultures is reduced at DIV35 and 56. (A)** Representative western blot images of iPSC-derived dopaminergic neuron samples at both DIV 35 and 56, with quantification for each protein normalised to β-actin **(B-I)**. Graphs show mean ± SEM from four controls, five *LRRK2-G2019S* lines and two *LRRK2-R1441C* lines, from three independent differentiations. Significance was assessed using a t-test between controls and *LRRK2-G2019S* lines only *p<0.05, **p<0.01.


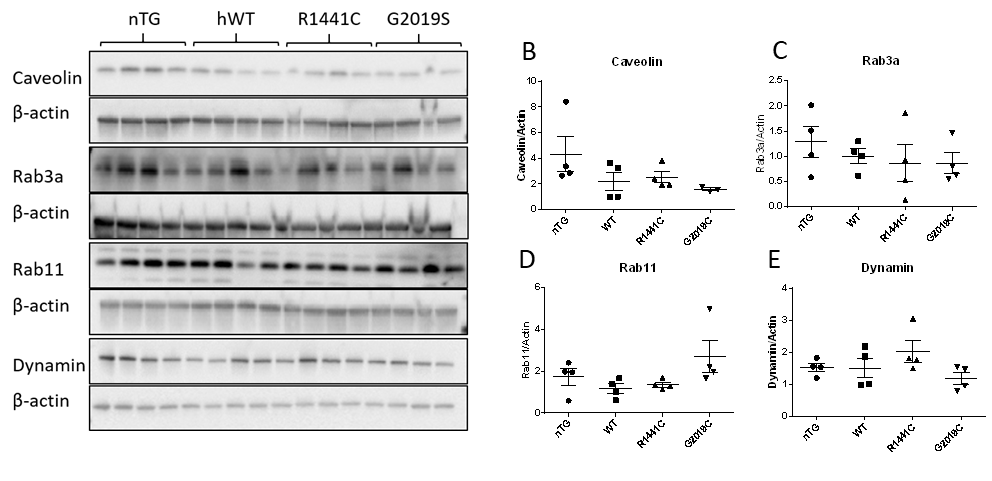


**Supplemental Figure S7**

**Endocytic proteins that are not differentially regulated by mutations in aged LRRK2 rats.**

Representative western blot images from 22-month old LRRK2 BAC transgenic rat striatal tissue **(A)** with quantification **(B-E)**. Graphs represent mean optical density normalised to β-actin ± SEM. One-way ANOVA.


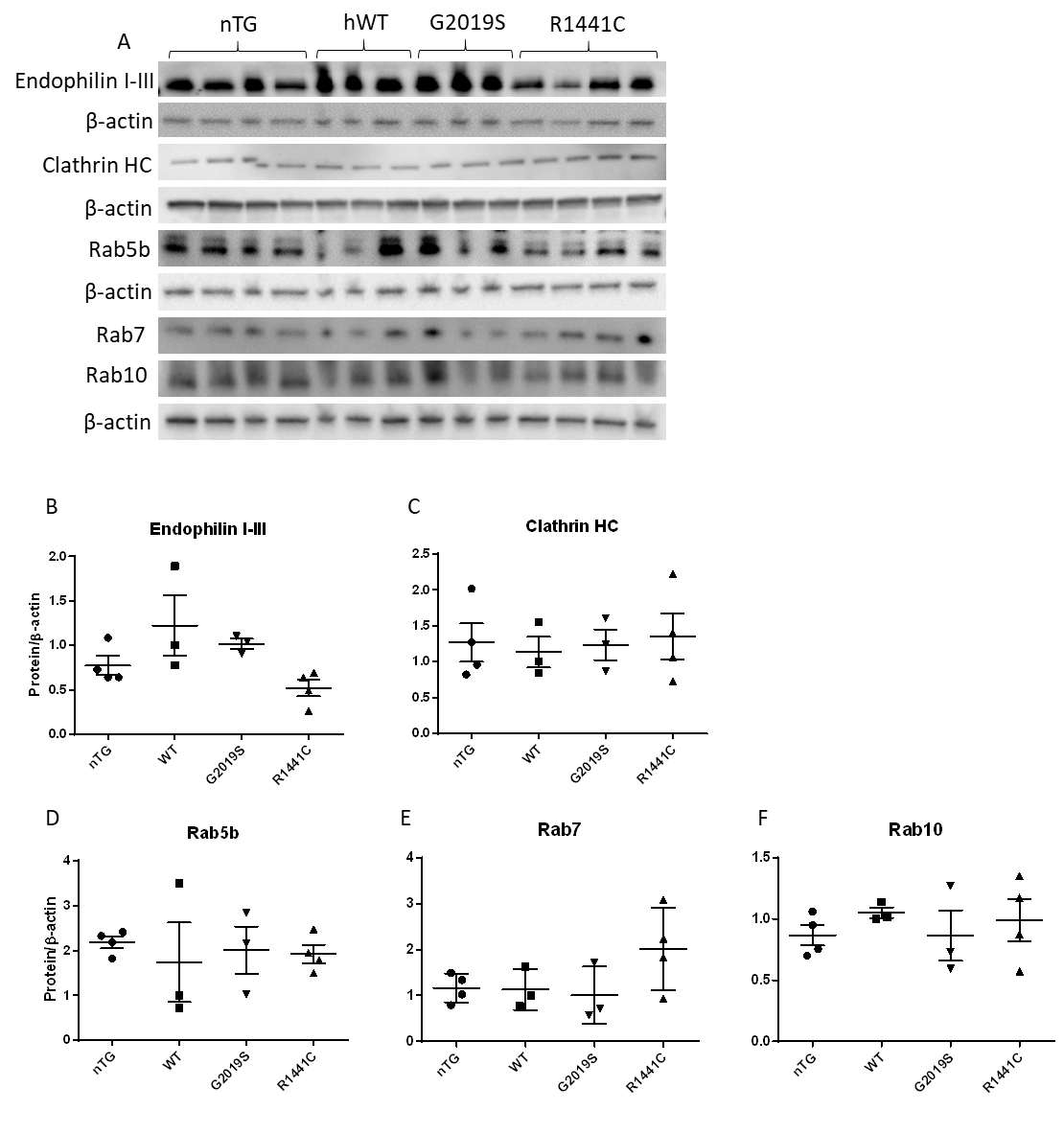


**Supplemental Figure S8**

**Endocytic proteins are unaltered in 12-month old LRRK2 rats.**

Representative western blot images from 12-month old LRRK2 BAC transgenic rat striatal tissue **(A)** with quantification **(B-F)**. Graphs represent mean optical density normalised to β-actin ± SEM. One-way ANOVA.

**
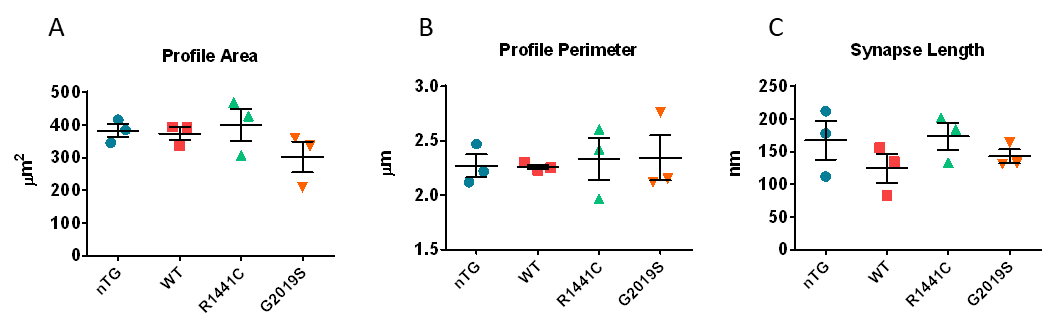
**

**Supplemental Figure S9**

**No gross morphological alterations are seen in dopaminergic terminals of LRRK2-R1441C and LRRK2-G2019S rats compared to controls.**

Graphs demonstrate no significant alterations in dopaminergic profile area **(A)**, profile perimeter **(B)** and synapse length **(C)** in the striatum of 22-month old rats expressing *LRRK2-R1441C* or *LRRK2-G2019S* in compared with controls. N=3 rats per genotype, 50 terminals assessed per rat. Graphs represent mean ± SEM. One-way ANOVA.


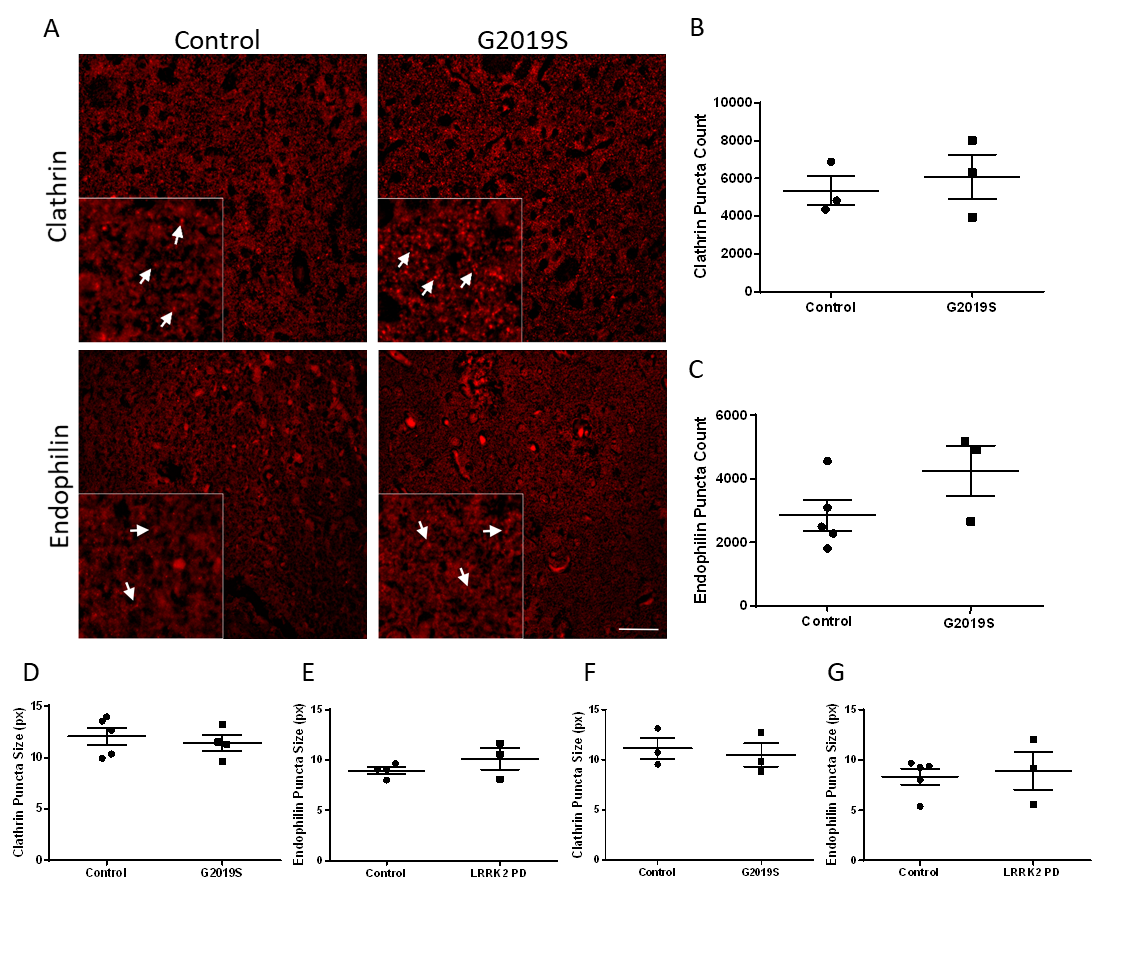


**Supplemental Figure S10**

**No changes in clathrin or endophilin puncta staining in human globus pallidus**

**(A)** Representative images of human globus pallidus tissue from healthy age matched controls and G2019S PS patients stained for either clathrin or endophilin, and quantification of puncta number **(B, C)**. Scale bar represents 50 µm. Quantification of puncta size for clathrin **(D)** and endophilin **(E)** from putamen samples and from globus pallidus **(F, G)**. Graphs show mean ± SEM. Unpaired t test; n=3-5 per group.
